# Association between malnutrition and cognitive decline in Chinese older adults: the mediating role of physical frailty

**DOI:** 10.3389/fpubh.2026.1843902

**Published:** 2026-06-15

**Authors:** Lian Li, Hongying Yang, Lujie Yu, Sa Xiao, Fengsheng Zhu

**Affiliations:** 1 Department of Psychiatry, Zhejiang Key Laboratory of Drug Addiction and Brain Health, Affiliated Kangning Hospital of Ningbo University (Ningbo Kangning Hospital), Ningbo, Zhejiang, China; 2 School of Public Health, Ningbo University Health Science Center, Ningbo University, Ningbo, Zhejiang, China

**Keywords:** cognitive decline, frailty, malnutrition, mediation analysis, older adults

## Abstract

**Background:**

Cognitive decline is a common neurodegenerative disorder in older adults. However, few studies have examined the interrelationships between malnutrition, physical frailty, and cognitive decline.

**Methods:**

A multi-stage cluster sampling approach was employed to recruit participants, namely older adults aged ≥65, from communities in Ningbo, China. The participants’ cognitive decline was evaluated using the Brief Screening Scale for Dementia. Malnutrition and physical frailty were assessed using the Mini Nutritional Assessment – Short Form and the Fatigue, Resistance, Ambulation, Illnesses, and Loss of Weight scale. Logistic regression was conducted to examine the association between malnutrition and cognitive decline, and mediation analysis was performed to assess the mediating role of physical frailty in this association. The validity of the association was tested by performing 1:1 nearest-neighbor propensity score matching (PSM).

**Results:**

The prevalence of malnutrition, physical frailty and cognitive decline were 17.04, 32.37 and 13.02%, respectively. The multivariate logistic regression indicated that malnutrition was significantly associated with physical frailty [odds ratio (OR): 1.45, 95% confidence interval (CI): 1.29–1.63] and cognitive decline (OR: 3.51, 95% CI: 3.06–4.03). Physical frailty was also significantly associated with cognitive decline (OR: 2.63, 95% CI: 2.31–3.00) and partially mediated the observed linkage between malnutrition and cognitive decline (*β* = 0.012, *p* < 0.001). The abovementioned results were confirmed by the data analysis performed after PSM.

**Conclusion:**

This study found that there was an association between malnutrition and cognitive decline in a sample of community-dwelling older adults in Ningbo, China, and that this association was mediated by physical frailty.

## 1 Introduction

The average human lifespan has steadily increased due to improvements in medical and healthcare conditions in most countries (1), leading to progressively aging global populations that have become a major societal challenge worldwide (1). China has now transitioned into a moderately aging society, as its population aged 65 and older reached 220 million in 2024 (2). In addition, the accelerating aging of China’s population means that geriatric syndromes, such as physical frailty and cognitive decline, have become a critical public health problem.

Cognitive impairment, including mild cognitive impairment (MCI) and dementia, is a common neurodegenerative disorder among older adults. Its early stage is characterized by significant memory decline accompanied by impairments in daily living and social activities. Moreover, the prevalence of cognitive impairment increases significantly with advancing age (3). In China, approximately 14.7% of adults aged 60 and older are affected by MCI (3). In addition, the economic burden of Alzheimer’s disease (AD) in China is currently estimated to be US$167.74 billion and is projected to rise to US $2,540 billion by 2030 (4). Furthermore, no pharmacological interventions effectively prevent or treat cognitive impairment (5). Hence, a critical focus of current research is to identify modifiable risk factors for cognitive impairment that will enable it to be prevented or delayed, thereby addressing societal challenges associated with population aging.

Good nutrition is essential for maintaining health and supporting development. However, malnutrition is a prevalent health problem among older adults (6). In China, nearly 35% of subjects were found to exhibit mild or moderate-to-severe malnutrition (7). Moreover, malnutrition in older adults in China (8), the United States of America (9), and Greece (10) was found to be associated with decreased cognitive performance. Similarly, a meta-analysis of nine studies determined that older adults with malnutrition had an increased risk of subsequent cognitive impairment (11). Suboptimal nutritional status, such as micronutrient deficiencies and protein–energy undernutrition, contributes to physical functional decline in older adults (12), which may be associated with their cognitive function decline. Relatedly, a 2-year follow-up study of non-demented Japanese older adults revealed that physical frailty was significantly associated with global cognitive function decline (13). Furthermore, a study of older adults in the USA found that both pre-physical frailty and physical frailty were associated with decreased cognitive performance (14). An eight-year follow-up of community-dwelling older Chinese adults revealed that physical frailty correlates with cognitive decline, especially in individuals with persistent or progressively worsening frailty status (15).

Existing local studies have validated the correlations of malnutrition-cognitive decline and frailty-cognitive decline, respectively. Nonetheless, most prior works seldom adopt propensity score matching to balance baseline differences, and rarely explore the mediating linking malnutrition, physical frailty and cognitive dysfunction. Thus, this study assessed the cognitive function status of older adults in Ningbo, investigated the association of cognitive function with nutritional status, and analyzed the mediating role of physical frailty in this association, with the aim of providing evidence for preventing cognitive impairment in the aging population.

## 2 Materials and methods

### 2.1 Study design and population

Ningbo, located on the southern wing of the Yangtze River Delta in eastern China, is a typical economically developed city with a rapidly aging population. In 2022, it had a population of approximately 9.6 million, including 1.4 million people aged 65 and above. The data for this study were derived from a cross-sectional study conducted in Ningbo. Between April and December 2024, a cross-sectional survey intended to assess cognitive function was conducted using a multi-stage cluster sampling method among community-dwelling older adults in Ningbo. The sampling process comprised three stages. First, five districts or counties (Fenghua District, Jiangbei District, Zhenhai District, xiangshan County, and Ninghai County) were selected using systematic sampling with probability proportional to size, representing half of all the administrative districts in Ningbo. Second, one street or township was randomly selected from each chosen district or county. Third, one community was randomly selected from each sampled street or township. The inclusion criteria were residence in Ningbo for at least one year, age ≥65 years, agreement to participate, and no clinical diagnosis of AD or other cognitive impairments. The exclusion criterion was an inability to provide signed informed consent or complete the questionnaire survey. A total of 10,530 individuals were initially approached to participate in the survey, and 378 participants were excluded due to being under 65 years of age. Data were collected via a digital survey platform, with all participants signing either electronic or paper-based informed consent forms. The study was approved by the Ethics Committee of Affiliated Kangning Hospital of Ningbo University (NBKNYY-2024-LC-10).

### 2.2 Assessment of cognitive function

The Chinese version of the 30-item Brief screening Scale for Dementia (BSSD) was used to assess cognitive function (16). Developed by Zhang Mingyuan, the BSSD is a short cognitive screening tool with high sensitivity and specificity and consists of six cognitive domains: common sense and image understanding (6 points), short-term and instant memory (6 points), attention and calculation (3 points), location and time targeting (9 points), language (3 points), and naming ability (3 points). Cronbach’s alpha for the BSSD in Chinese older adults was 0.88 (16). In Chinese older adults with cognitive decline were defined as those with BSSD scores ≤ 18 points (for participants who were illiterate), ≤ 21 points (for those with an elementary-school education), and ≤ 24 points (for those with a middle-school education or higher) (16).

### 2.3 Assessment of nutritional status

Nutritional status was assessed using the Mini Nutritional Assessment – Short Form (MNA-SF) (17), which consists of six items: decrease in food intake (2 points), weight loss during last three months (3 points), mobility (2 points), acute health status (psychological stress or acute disease) (2 points), neuropsychological problems (dementia or depression) (2 points), and body mass index (3 points). In a previous study, older adults with MNA-SF scores of 0–7, 8–11, and 12–14 points were considered to have malnutrition, be at risk of malnutrition, and have a good nutrition status, respectively (17). Considering the limited sample size of individuals with confirmed malnutrition, we combined participants with malnutrition and those at nutritional risk into a poor nutrition status group for subsequent analysis (17).

### 2.4 Assessment of physical frailty

Physical frailty was assessed using the Fatigue, Resistance, Ambulation, Illnesses, and Loss of Weight (FRAIL) scale, which comprises five domains: fatigue, resistance, ambulation, illnesses, and loss of weight (18). Older adults with FRAIL scores of 0, 1–2, or 3–5 were regarded as robust, pre-frail, or frail, respectively. Given the relatively small sample of frail participants in this study, pre-frail and frail cases were combined into a unified physical frailty group, consistent with prior research (18).

### 2.5 Covariates

The covariates were the following sociodemographic characteristics: age, sex, education level (<6 or ≥6 years), residence (urban or rural), marital status (married or other), smoking status (yes or no), alcohol-drinking status (yes or no), hearing loss (yes or no), comorbidity (yes or no), sleep quality (poor or good), physically active (yes or no), loneliness (yes or no), social isolation (yes or no), and depression (yes or no) (19). Depression was assessed using the nine-item Patient Health Questionnaire, with scores of at least 10 regarded as indicating the presence of depressive symptoms (19).

### 2.6 Statistical analysis

Differences in quantitative variables with a normal distribution are reported as means ± standard deviations and were analyzed by performing *t* tests. Differences in categorical variables are presented as numbers (percentages) and were analyzed by performing chi-square tests. After unadjusting and adjusting for covariates, we conducted a logistic regression analysis to examine the associations between malnutrition, physical frailty, and cognitive decline. The results are presented as odds ratios (ORs) with 95% confidence intervals (CIs). Bootstrapping was used to assess the significance of the total, indirect, and direct utility of the mediation model (20). The direct relationship between malnutrition and cognitive decline and the mediating effect of physical frailty were calculated. The analysis involved 100 bootstrap resamples, and the effect size was assessed using a nonparametric percentile bootstrap CI; a 95% CI excluding zero indicated statistical significance (21). One-to-one nearest-neighbor propensity score matching (PSM) was used to mitigate residual confounding and balance baseline characteristics across the case and control groups, with age, sex, education level, residence, marital status, smoking status, alcohol-consumption status, hearing loss, comorbidity, et al. as confounding factors for matching. Following PSM, the sample was re-analyzed to further test the validity of the results. A *p* value of less than 0.05 was considered to indicate statistical significance. Data processing and analysis were performed using R version 4.4.3 and Zstats v1.0 (www.zstats.net).

## 3 Results

The study sample comprised 10,152 older adults, of whom 4,511 (44.43%) were male and 1,322 (13.02%) were classified as having cognitive decline. As shown in Table 1, compared with the group with cognitive decline, the group without cognitive decline had a large proportion of men, a lower education level, married, smoking, drinking, a lower level of sleep quality, physical activity. Additionally, the proportions of participants with comorbidity, hearing loss, isolation, and loneliness were higher in the group with cognitive decline than the group without cognitive decline (*p* < 0.001). After PSM, there were no significant between-group differences in terms of sex, education level, urban vs. rural residence, smoking status, alcohol-consumption status, hearing loss, comorbidity, physical activity, loneliness, or depression (all *p* > 0.05, Table 1).

**Table 1:** Sample characteristics of the study population by cognitive function before and after PSM.

Characteristics	Before PSM				After PSM			
	Cognitive decline		SMD	*P*	Cognitive decline		SMD	*P*
Variables	No (*n* = 8,830)	Yes (*n* = 1,322)			No (*n* = 1,289)	Yes (*n* = 1,289)		
Age, Mean ± SD	72.36 ± 5.49	75.92 ± 6.52	0.545	<0.001	75.58 ± 6.50	75.65 ± 6.32	0.011	0.775
Sex, *n* (%)			0.008	0.007			0.057	0.152
Male	3,969 (44.95)	542 (41.00)			567 (43.99)	531 (41.19)		
Female	4,861 (55.05)	780 (59.00)			722 (56.01)	758 (58.81)		
Education year, *n* (%)			0.247	<0.001			0.077	0.053
< 6 years	6,914 (78.30)	881 (66.64)			808 (62.68)	855 (66.33)		
≥ 6 years	1916 (21.70)	441 (33.36)			481 (37.32)	434 (33.67)		
Residence, *n* (%)			0.198	<0.001			0.029	0.471
Urban	5,906 (66.89)	997 (75.42)			950 (73.70)	966 (74.94)		
Rural	2,924 (33.11)	325 (24.58)			339 (26.30)	323 (25.06)		
Marital status, *n* (%)			0.203	<0.001			0.029	0.460
Married	7,885 (89.30)	1,076 (81.39)			1,080 (83.79)	1,066 (82.70)		
Others	945 (10.70)	246 (18.61)			209 (16.21)	223 (17.30)		
Smoking, *n* (%)			0.084	0.007			0.014	0.719
No	6,920 (78.37)	1,079 (81.62)			1,061 (82.31)	1,054 (81.77)		
Yes	1910 (21.63)	243 (18.38)			228 (17.69)	235 (18.23)		
Drinking, *n* (%)			0.100	0.002			0.005	0.903
No	7,502 (84.96)	1,166 (88.20)			1,136 (88.13)	1,134 (87.98)		
Yes	1,328 (15.04)	156 (11.80)			153 (11.87)	155 (12.02)		
Hearing loss, *n* (%)			0.271	<0.001			0.042	0.280
No	8,169 (92.51)	1,086 (82.15)			1,096 (85.03)	1,076 (83.48)		
Yes	661 (7.49)	236 (17.85)			193 (14.97)	213 (16.52)		
Comorbidity disease, *n* (%)			0.120	<0.001			0.029	0.458
No	3,053 (34.58)	385 (29.12)			359 (27.85)	376 (29.17)		
Yes	5,777 (65.42)	937 (70.88)			930 (72.15)	913 (70.83)		
Sleep quality, *n* (%)			0.088	0.003			0.096	0.015
Good	6,003 (67.98)	952 (72.01)			867 (67.26)	1791 (71.67)		
Poor	2,827 (32.02)	370 (27.99)			422 (32.74)	365 (28.32)		
Physical activity, *n* (%)			0.086	0.004			0.005	0.901
No	5,504 (62.33)	878 (66.41)			844 (65.48)	847 (65.71)		
Yes	3,326 (37.67)	444 (33.59)			445 (34.52)	442 (34.29)		
Social isolation, *n* (%)			0.134	<0.001			0.095	0.011
No	7,941 (89.93)	1,126 (85.17)			1,146 (88.91)	1,103 (85.57)		
Yes	889 (10.07)	196 (14.83)			143 (11.09)	186 (14.43)		
Loneliness, *n* (%)			0.213	<0.001			0.071	0.062
No	7,887 (89.32)	1,070 (80.94)			1,096 (85.03)	1,061 (82.31)		
Yes	943 (10.68)	252 (19.06)			193 (14.97)	228 (17.69)		
Depression, *n* (%)			0.174	<0.001			0.035	0.339
No	8,804 (99.71)	1,276 (96.52)			1,272 (98.68)	1,266 (98.22)		
Yes	26 (0.29)	46 (3.48)			17 (1.32)	23 (1.78)		
Nutrition Status, *n* (%)			-	<0.001			-	<0.001
Good	7,628 (86.39)	794 (60.06)			1,072 (83.17)	787 (61.06)		
Poor	1,202 (13.61)	528 (39.94)			719 (16.83)	719 (38.94)		
Physical frailty, *n* (%)			-	<0.001			-	<0.001
No	6,277 (71.09)	589 (44.55)			809 (65.76)	586 (45.46)		
Yes	2,553 (28.91)	733 (55.45)			480 (37.24)	703 (54.34)		

PSM, propensity score matching.

The prevalence of malnutrition and physical frailty were 17.04 and 32.37%, respectively (Table 1). Compared with the group without cognitive decline, the group with cognitive decline had a significantly higher prevalence of malnutrition (39.94% vs. 13.61%, *p* < 0.001) and physical frailty (55.45% vs. 28.91%, *p* < 0.001).

In the multivariate logistic regression, compared with those without malnutrition, those with malnutrition were 1.45 times and 1.24 times more likely to exhibit physical frailty before and after PSM, respectively (both *p* < 0.001, Table 2).

**Table 2:** The associations between malnutrition and physical frailty.

Characteristics	OR^a^(95% CI)	OR^b^(95% CI)
Before PSM		
Malnutrition	1.48 (1.33 ~ 1.65)	1.45 (1.29 ~ 1.63)
After PSM		
Malnutrition	1.09 (0.92 ~ 1.29)	1.24 (1.02 ~ 1.50)

^a^The model was unadjusted for confounding factors. ^b^The model was adjusted for age, sex, residence, education year, marital status, drinking status, smoking status, hearing loss, comorbidity disease, sleep quality, physical activity, loneliness, social isolation, depression. PSM, propensity score matching.

In the multivariate logistic regression, compared with those without physical frailty, those with physical frailty were 2.63 times and 2.34 times more likely to experience cognitive decline before and after PSM, respectively (both *p* < 0.001, Table 3).

**Table 3:** The associations between malnutrition, physical frailty and cognitive decline.

Characteristics	OR^a^(95% CI)		
	Model 1	Model 2	Model 3
Before PSM			
Malnutrition	3.51 (3.06 ~ 4.03)	—	3.37 (2.93 ~ 3.88)
Physical frailty	—	2.63 (2.31 ~ 3.00)	2.51 (2.20 ~ 2.87)
After PSM			
Malnutrition	3.34 (2.76 ~ 4.05)	—	3.35 (2.76 ~ 4.08)
Physical frailty	—	2.34 (1.97 ~ 2.78)	2.36 (1.97 ~ 2.82)

^a^The model was adjusted for age, sex, residence, education year, marital status, drinking status, smoking status, hearing loss, comorbidity disease, sleep quality, physical activity, loneliness, social isolation, depression, malnutrition or physical frailty. PSM: propensity score matching.

The model without physical frailty showed that those with malnutrition were 3.51 times more likely to experience cognitive decline than those without malnutrition (OR: 3.51, 95% CI: 3.06–4.03; Table 3). In addition, this model with physical frailty showed that malnutrition was positively associated with an increased likelihood of cognitive decline (OR: 3.37, 95% CI: 2.93–3.88; Table 3). After PSM, this positive association persisted.

**Figure 1:**
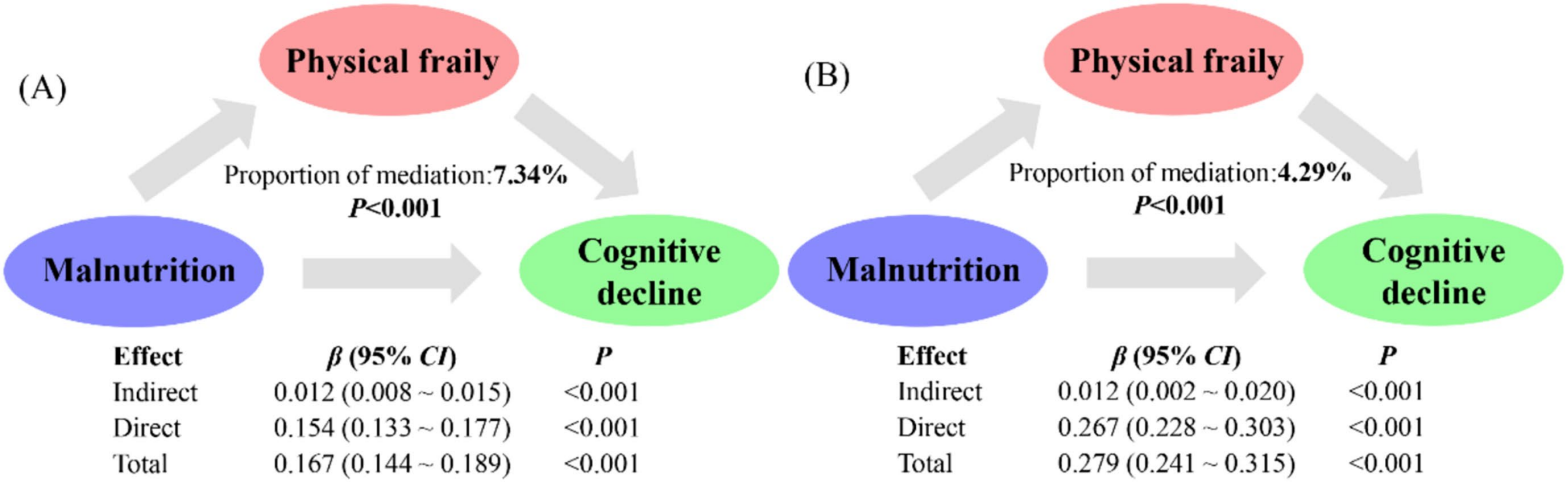
Mediation analysis for physical fraily in the association between malnutrition and cognitive decline. **(A)** Before PSM, **(B)** After PSM.

As shown in Figure 1, mediation analysis revealed that physical frailty was an independent mediator of the detrimental effect of malnutrition on cognitive decline. Specifically, 7.34 and 4.29% of this effect was mediated through physical frailty before and after PSM, respectively.

## 4 Discussion

This study was the first to examine whether physical frailty mediated an association between malnutrition and cognitive decline in a sample of community-dwelling older adults. The prevalence of cognitive decline in our sample of community-dwelling older adults in Ningbo, China, was 13.02%, whereas it was 10.97% in the 2018 Chinese Longitudinal Healthy Longevity Survey (22). Moreover, following covariate adjustment, malnutrition remained significantly correlated with physical frailty and cognitive decline in analyses conducted before and after PSM. Furthermore, the effect of malnutrition on cognitive decline was partially mediated by physical frailty. In the analysis, PSM balanced intergroup baseline disparities to reduce selection bias. Subsequent covariate adjustment further excluded confounding interference.

Nutritional status plays a crucial role in the development of physical frailty. For example, in older adults, malnutrition may lead to decreased physical activity, chronic inflammation, and vitamin D deficiency (6), all of which contribute to the onset of physical frailty. In addition, malnutrition is highly prevalent among older adults (6) and is a primary risk factor for sarcopenia (23), which is a manifestation of physical frailty. Thus, ensuring an adequate and balanced intake of macronutrients and micronutrients to maintain optimal nutritional status can effectively slow the progression of physical frailty. Kobayashi et al. (24) conducted a multicenter study involving 2,108 Japanese women aged 65 and older and demonstrated a significant association between their total protein intake and physical frailty status. Similarly, Qin et al. (25) studied a sample of older adults in China and identified a strong association between malnutrition and physical frailty, which can serve as the basis for developing a risk prediction model for physical frailty.

Our results show that compared with participants without physical frailty, those with physical frailty tended to have more impaired cognitive function, which is consistent with findings from previous studies (26). These findings may be attributable to the fact that reduced muscle mass in physically frail older adults leads to sarcopenia, which is associated with an increased risk of cognitive impairment (26). In addition, inflammation plays a significant role in the association between physical frailty and cognitive decline. Specifically, communication and interactions between the central nervous system and the immune system are dynamic and continuous (27). In older adults with physical frailty, increased concentrations of peripheral inflammatory markers contribute to increased inflammation within the central nervous system (27). These centrally acting inflammatory factors are also neurotoxic and may consequently impair cognitive function (28). In addition, a deficiency of androgens, such as testosterone, may be involved in the relationship between physical frailty and cognitive decline (29).

The results of this cross-sectional study show that the prevalence of cognitive impairment among malnourished participants was higher than that among non-malnourished participants. Moreover, a cross-sectional study conducted in China indicates that malnutrition is significantly associated with an increased risk of cognitive dysfunction in the participants (8). Similarly, Albala et al. (30) studied 2,372 older adults (aged > 60) in Chile and found that after 10–15 years of follow-up, the incidence of cognitive dysfunction among those in the physical frailty group was 48.1%, significantly higher than that among older adults in the non-frailty group (20.5%). The aforementioned findings may be primarily related to the “gut–brain axis” (31). This theoretical axis is a sophisticated bidirectional communication system connecting the gastrointestinal tract to the central nervous system. Gut microbiota exert modulatory effects on cognitive functions through this axis via multiple mechanisms, such as microbial metabolite production, vagus nerve signaling, and immune pathway mediation (31, 32). As older adults age, their nutrient intake, metabolism, and absorption become insufficient (33), causing an imbalance in their gut microbiota. Thus, early screening and timely diagnosis of both malnutrition and physical frailty are essential for preventing physical and cognitive functional decline in older adults.

This study indicated physical frailty partially explains the association between malnutrition and cognitive decline. Nutritional status correlates with cognitive function among community-dwelling older adults through direct pathways as well as indirect pathways mediated by physical frailty. This mediating effect was found to account for 7.34% of the total effect. A study of older adult patients (aged > 80) in a hospital in China showed that physical frailty mediated the association between nutrition and cognitive function, with the mediation effect accounting for 41.5% of the total effect (34). This higher mediation effect relative to the current study might be due to the older age and inpatient nature of the previous study’s population. Accumulated evidence confirms that physical frailty mediates the association between malnutrition and cognitive decline in older adults. This systemic degenerative syndrome encompasses far more than isolated muscle weakness, presenting with slowed gait, reduced activity tolerance and diminished physiological resilience (18, 26). Nutritional deficiency undermines overall physical function, disrupts cerebral metabolism and neural activity, and thereby exacerbates frailty and subsequent cognitive impairment (34).

Due to older adults’ declining physical function and changing dietary structure, their bodies lack many proteins and nutrients (6). Thus, older adults exhibit a high incidence of malnutrition. Therefore, great importance should be attached to encouraging older adults to consume a diet that provides adequate nutrition. These findings may offer potential reference for clinical practice. Medical staff can conduct regular nutritional-risk assessments of older adults and subsequently formulate individualized nutritional support plans for them. In addition, medical staff can establish a multidisciplinary team to ensure that older adults undergo physical frailty and cognitive screening and other comprehensive assessments.

### 4.1 Strengths and limitations

This is the first study to explore how physical frailty mediates the association between malnutrition and cognitive decline in a sample of community-dwelling older adults in China. Thus, our findings provide a new perspective on the prevention and control of cognitive decline in such populations. Nonetheless, this study has some limitations. First, the participants were members of the older adult population in Ningbo, China. Therefore, due to differences in races, economies, and cultures, our conclusions are not generalizable to other populations. Second, key variables, such as nutritional status, physical frailty, and cognitive function, were assessed using previously designed scales, and although their effectiveness has been verified, our results might have been affected by information and reporting bias. Third, cross-sectional studies cannot establish causal relationships, meaning that longitudinal studies are needed to establish temporal relationships. Residual confounding may persist even after thorough covariate adjustment. Frailty, a multidimensional physiological condition closely associated with multimorbidity and reduced physiological reserve, could affect identified correlations (35). Additionally, the concise screening tool cannot fully reflect clinical complexity of frailty, limiting the interpretation of study findings.

## 5 Conclusion

This study explored the association between malnutrition and declining cognitive function in a sample of community-dwelling older adults in Ningbo, China, and whether this association was mediated by physical frailty. The findings suggest targeted interventions to mitigate malnutrition and frailty may benefit health management for community-dwelling older adults in China. To this end, early screening of this population for malnutrition, physical frailty, and cognitive impairment is needed to effectively alleviate the burden of cognitive disorders.

## Data Availability

Publicly available datasets were analyzed in this study. This data can be found here: data cannot be made available online due to legal and ethical restrictions and will be made available by LL (ll19857840971@163.com) upon reasonable request.
